# Cerebral palsy and sleep: nonpharmacological treatment and impact on the life of caregivers – an integrative review

**DOI:** 10.1055/s-0044-1781464

**Published:** 2024-03-11

**Authors:** Marcela Fischer de Almeida, Suzane Mello, Marise Bueno Zonta, Ana Chrystina Crippa

**Affiliations:** 1 Universidade Federal do Paraná, Curitiba PR, Brazil.

**Keywords:** Cerebral Palsy, Caregivers, Sleep, Child, Paralisia Cerebral, Cuidadores, Sono, Criança

## Abstract

**Background** Children with cerebral palsy have a higher prevalence of sleep disorders, with numerous factors associated with a negative impact on the quality of life of caregivers.

**Objective** To identify factors related to sleep disorders, nonpharmacological treatment, and the impact on the lives of caregivers.

**Methods** The present literature review was carried out in the Latin American and Caribbean Center on Health Sciences Information (BIREME), the Cochrane Library, Scopus, PubMed, the Cumulative Index to Nursing and Allied Health Literature (CINAHL), PsycInfo, WorldCat, Web of Science, Latin American Literature on Health Sciences (LILACS), and Excerpta Medica Database (EMBASE), with the descriptors *sleep, child, cerebral palsy, parents* , and *nursing* . Studies available in Portuguese, English, or Spanish, published between 2010 and 2020, were our inclusion criteria. A total of 29 articles were included in the present review.

**Results** We considered nonpharmacological interventions effective support measures to drug-based treatments. The main sleep disorders in children with cerebral palsy are insomnia, parasomnias, nightmares, sleep bruxism, sleepwalking, sleep talking, disorders of initiation and maintenance of sleep, and sleep hyperhidrosis. Most studies point to a reduction in the quality of life of caregivers whose children have sleep disorders.

**Conclusion** Our review suggests the effectiveness of nonpharmacological treatments combined with the use of medications. Measures such as changes in sleep environment and routine are favorable strategies to improve sleep quality. In addition, children with sleep disorders negatively impact the quality of life of their caregivers.

## Resumo

**Antecedentes** Crianças com paralisia cerebral apresentam maior prevalência de distúrbios do sono, com inúmeros fatores associados a um impacto negativo na qualidade de vida dos cuidadores.

**Objetivo** Identificar fatores relacionados aos distúrbios do sono, o tratamento não farmacológico e o impacto na vida dos cuidadores.

**Métodos** Esta revisão da literatura foi realizada no Centro Latino-Americano e do Caribe de Informação em Ciências da Saúde (BIREME), Biblioteca Cochrane, Scopus, PubMed, Cumulative Index to Nursing and Allied Health Literature (CINAHL), PsycInfo, WorldCat, Web of Science, Literatura Latino-Americana em Ciências da Saúde (LILACS) e Excerpta Medica Database (EMBASE), com os descritores *sono, criança, paralisia cerebral, pais* e *enfermagem* . Estudos disponíveis em português, inglês ou espanhol, publicados entre 2010 e 2020, foram nossos critérios de inclusão. Ao todo, 29 artigos foram incluídos nesta revisão.

**Resultados** Consideramos as intervenções não farmacológicas medidas eficazes de apoio aos tratamentos medicamentosos. Os principais distúrbios do sono em crianças com paralisia cerebral são: insônia, parassonias, pesadelos, bruxismo do sono, sonambulismo, falar dormindo, distúrbios de iniciação e manutenção do sono e hiperidrose do sono. A maioria dos estudos aponta redução na qualidade de vida de cuidadores de crianças com distúrbios do sono.

**Conclusão** Nossa revisão sugere a eficácia de tratamentos não farmacológicos combinados com o uso de medicamentos. Medidas como mudanças no ambiente e na rotina do sono são estratégias favoráveis para melhorar a qualidade do sono. Além disso, crianças com distúrbios do sono provocam impactos negativos na qualidade de vida de seus cuidadores.

## INTRODUCTION

Cerebral palsy (CP) refers to “a group of permanent movement and posture disorders, causing activity limitations” 1 . According to Graham et al. 2 , CP is divided into five types: diplegia, hemiplegia, quadriplegia, dyskinesia, and ataxia. Cerebral palsy is often followed by sensory, communication, perception, cognition, and behavior impairments, as well as by other diseases, such as epilepsy and secondary musculoskeletal problems, and, in some cases, by sleep disorders (SDs). 2 3 4

According to recent studies, 5 6 there is a high prevalence of SDs in children with CP, with rates ranging from 23.4% to 46%. Motor impairment in CP is also a factor that interferes in the quality of sleep due to muscle spasms, contractures, and decreased ability to change body position during the night. 5 7

Furthermore, various environmental factors can also contribute to SDs in children, such as visual environment, voices, sounds, smells, and an unfamiliar bed. Sleep habits such as bedtime routines, daily caffeine intake, and sleep environment (temperature) may also contribute. 7 8

The impaired sleep of children with CP and the night care required by them can affect the health of caregivers. This is a relevant aspect to be studied because high levels of stress caused by emotional, social, and personal issues can be exacerbated in the presence of sleep deprivation, and this will consequently impact the quality of life of caregivers. 9 10 11

Finally, in children with CP, SDs can impact their quality of life. In this group, quality of life is linked to the motor, emotional, social, and school commitments, which suffer the effect of impaired sleep. 7 12 Therefore, it is extremely significant to better understand sleep management in these individuals. In summary, the present integrative review aims to identify the factors for SDs in children with CP and to present some nonpharmacological intervention approaches, in addition to showing how this disorder negatively impacts the quality of life of caregivers.

### Study questions and purpose

The objective of the present integrative review is to provide an extensive understanding and synthesis of the scientific literature on the nature of SDs in children with CP, the factors related to this disorder, its treatment, and its impact on the lives of caregivers. The research questions were: What are the nature and factors associated with SDs in children with CP? What are the interventions for these disorders and what is the impact on the lives of caregivers?

## METHODS

The integrative review approach is a research method in which a summary of the literature on a particular topic is conducted for its better understanding and comprehension. 7 In contrast with systematic reviews, which include only quantitative studies, this method includes all studies that meet the criteria of a rigorous selection process of articles. 7 This model of research is useful to aggregate information from a set of studies conducted separately, with the objective of collaborating with the construction of science, evidence-based practice, and public policy making. 13 In addition, it is possible to identify topics that have a low level of evidence coverage and thus guide future research. We selected articles that elucidate the already consolidated knowledge in the international literature on the general prevalence of the main SDs, as well as their associated factors and nonpharmacological interventions in sleep alterations. Given these data, it is possible to analyze the study trends, the methodologies used in the last ten years, and how impactful are the advances in this area. The review included articles published in the last10 years to analyze the most recent findings in the literature.

For this purpose, 2 researchers conducted an independent systematic search, from March to May 2020, using the following databases in sequence: Latin American and Caribbean Center on Health Sciences Information (BIREME), the Cochrane Library, Scopus, PubMed, the Cumulative Index to Nursing and Allied Health Literature (CINAHL), PsycInfo, WorldCat, Web of Science, Latin American Literature on Health Sciences (LILACS), and Excerpta Medica Database (EMBASE). The search terms were: *sleep, child, cerebral palsy, caregiver, parents* and *nursing* . Each subsequent stage of the review was conducted independently by two researchers (MFA and either SM or MBZ) to reduce bias, with disputes raised with a third member of the review team if necessary.

The inclusion criteria were studies that:

• were written in Portuguese, English or Spanish;
• addressed SDs in children diagnosed with CP, aged between 0 and18 years;
• were published between 2010 and 2020;
• analyzed the prevalence of SDs;
• examined the factors associated with SDS in children with CP;
• investigated the influence (impact) on the family and caregivers of this group of interest; and
• analyzed the main nonpharmacological interventions in CP SDs.

### The exclusion criteria were studies that:

• were published as editorials or letters to the editor;
• were not published in peer-reviewed journals (such as abstracts or dissertations);
• appeared repeatedly in subsequent databases;
• were pilot studies with no published results; and
• were research in which SDs and/or their impact on the life of caregivers had been evaluated in a general category of neuromotor disorders, without a specific analysis for CP.

Articles with missing information met the exclusion criteria. The protocol for the present integrative review was not registered.

### Criteria to consider studies for the review

From the eligible articles, data was extracted regarding the sample characteristics (number of CP patient/caregiver participants, details of control groups, recruitment site, age, gender, and main nonpharmacological interventions), study methods, and results. The data extracted from each study included the following:

• Study design (such as identification of research questions, a literature search, categorization and assessment of studies, interpretation of results, and synthesis of knowledge).
• Participant details (such as number, age, gender, diagnosis of CP, risk factors for sleep problems, and general data about caregivers).
• Sleep assessment measures (such as objective measures, questionnaires, and results of main analyses).
• Nonpharmacological intervention details (such as components of behavioral intervention, guidance to parents, and type of control group[s]).
• Caregiver outcome measures (such as objective and self-reported measures of mood and behavior, and results of main analyses).

### Assessment of risk bias in included studies

Pairs of review authors independently assessed the risk of bias of the included studies. One review author (MFA or SM) checked consistency in assessment at data entry. We used the tool outlined in the Preferred Reporting Items for Systematic Reviews and Meta-Analyses (PRISMA) statement. 14 We extracted details on the study population, study environment, intervention, study methodology, and outcomes of each study in order to enable quality appraisal, evaluation of external validity, and data analysis. We resolved any discrepancies in quality ratings through discussion and the involvement of an arbitrator when necessary.

### Quality appraisal and synthesis

The included articles were assessed for their methodological quality according to Melnyk and Fineout-Overholt . 15 The articles were classified according to the level of evidence: level I – systematic review or meta-analysis of randomized controlled trials or clinical guidelines based on systematic review of randomized controlled trials; level II – randomized controlled trials; level III – controlled trial without randomization; level IV – case-control and cohort studies; level V – systematic descriptive review and qualitative studies; level VI – descriptive or qualitative studies (single descriptive or qualitative studies); and level VII – opinion of authors and/or report of expert committees. 7 15

## RESULTS

**Figure 1:**
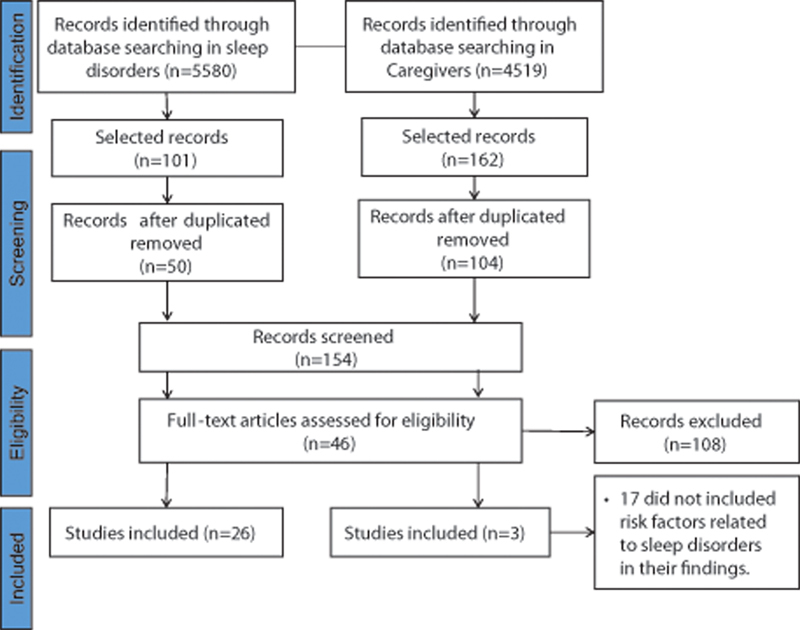
PRISMA flow diagram of the selection of studies.

Figure 1 shows the PRISMA flow diagram for the present review. The search in the online databases resulted in 5,580 studies related to SDs in CP, and 4,519 related to caregivers. We considered 154 studies potentially relevant, being 46 of which were pieces of research on SDs, 2 were articles about caregivers, and 1 was an article that had analyzed both SDs in children and their influence on the lives of caregivers. The complete description of each of the studies included and their characteristics can be found in the main text of the Results section and in Tables 1 and 2 .

**Table 1:** Summary of reviews on sleep disorders in cerebral palsy

**Study design**	Sample	Results	Type of sleep disorder	Reference
Systematic review and meta-analysis	23 cross-sectional studies with samples ranging from 50 to 675 children	15.5% had sleep breathing disorders; 14.6% had disorders of excessive somnolence; and 34.9% had sleep hyperhidrosis	Parasomnias; insomnia; restless leg syndrome	Horwood et al. 9
Systematic review	66 studies	Delayed sleep phase syndrome; impaired sleep maintenance	Obstructive sleep apnea	Galland et al. 36
Integrative review	1,385 individuals in the totality of studies	50% had sleep-disordered breathing; 50% had nightmares; 12.5% regularly talked in their sleep; and 62.5% had excessive daytime	Restless leg syndrome; obstructive sleep apnea; sleep bruxism; insomnia	Lélis et al. 7
Systematic review	40 international medical data studies	62.5% had difficulty in the initiation of sleep; 55% had bedtime resistance; and 57.5% had night-waking	Insomnia	Rigney et al. 47
Systematic review based on 2 randomized clinical trials	21 children	Not available	Obstructive sleep apnea	Blake et al. 30
Literature review	Not available	50% had sleep-disorderd breathing; and 50% had nightmares	Insomnia; sleep bruxism	Dutt et al. 21

**Table 2:** Summary of original articles on sleep disorders in cerebral palsy (CP)

Study design	Sample	Results	Sleep measure	Reference
Cross-sectional study	227 children (69 control group + 23 with CP + 106 with epilepsy + 29 with CP and epilepsy)	100% had sleep-disordered breathing; and 91% had excessive daytime sleepiness	Pediatric sleep questionnaire; sleep disordered breathing scale	Ming et al. 24
Cross-sectional study	132 individuals (91 with CP + 41 control group)	7.3% had sleep-disordered breathing; 1.0% had excessive daytime sleepiness	Pediatric sleep questionnaire	Sandella et al. 12
Cross-sectional study	100 individuals with CP	62.5% had excessive daytime sleepiness; 50% had sleep- disordered breathing; 50% had nightmares; and 12.5% had sleep talking	Sleep questionnaire	Elsayed et al. 25
Cross-sectional study	165 children with CP	22% had disorders of initiating and maintaining sleep; 14% had sleep-breathing disorders; 15% had sleep–wake transition disorder; 10% had disorders of arousal; and 7% had sleep hyperhidrosis	Sleep Disturbance Scale for Children	Romeo et al. 3
Cross-sectional study	113 children with CP	45.1% had frequent twitching or jerking of legs/often changes position/kicks the covers off the bed; 39.8% had difficulty falling asleep at night; 32.7% had nighttime awakenings; and 34.5% awaked in the morning feeling tired	Sleep Disturbance Scale for Children	Horwood et al. 17

Our sample consisted of 7 reviews, 1 meta-analysis, 2 case reports, 18 cross-sectional studies, 2 pilot studies, and 1 prospective cohort. We found 17 studies on risk factors for SDs in children with CP and 8 related to nonpharmacological treatment. Most studies were published in journals on sleep and child development, while 15 were published in periodicals in the field of neurology. Most studies presented a low level of evidence, with 6 of them scoring at level V and 21 at level VI. Among the analyzed works, only three – one meta-analyses and two systematic reviews – were classified as having a high level of evidence (levels I or II). Out of the 29 analyzed articles, 6 studies had been conducted in Canada, 3, in Australia, 3, in the United States, 3, in the United Kingdom, 2, in Brazil, 2, in Italy, 1, in New Zealand, 1, in India, 1, in Iran, 1, in Egypt, 1, in Turkey, 1, in Malaysia, 1, in Uganda, 1, in Russia, 1, in China, and 1, in Switzerland.

A total of 27 studies evaluated sleep by including at least 1 of the following aspects: prevalence of the main SD; risk factors; and nonpharmacological interventions in SD. Three studies evaluated the impact of SDs on caregivers and their associated factors. One study addressed both the prevalence of SDs and their impact on the lives of caregivers.

The 29 studies evaluated included a total of 3,046 subjects (2,996 children and 577 caregivers). The children were divided into groups according to their development stages: infants (0 to 11.9 months old); young children (12 to 35 months old); preschoolers (3 to 5 years old); school-aged (6 to12 years old); and teenagers (13 to 18 years old). The school-aged group was the most commonly studied (21 studies), followed by preschoolers (13), young children (10), adolescents (80), and infants (5).

The SDs included insomnia, arousal disorder, 16 17 18 19 20 sleep disordered breathing, 18 19 20 21 22 23 symptoms associated with insomnia, 12 24 25 parasomnia 3 21 23 ^,^ and nightmares, 11 sleep bruxism, 25 26 sleep walking and sleep talking, 25 27 disorders of initiation and maintenance of sleep, 3 5 8 10 16 20 23 28 sleep hyperhidrosis, 3 16 17 20 23 29 disorders of excessive somnolence, 3 10 12 16 17 22 24 25 27 reflux and digestion problems, 27 pain, 1 8 28 sleep-wake transition disorders, 3 19 20 nocturnal convulsions, 11 29 skeletal muscle/postural limitations, 22 30 and nonrestorative sleep. 23

Among the 18 original studies on factors associated with SD and nonpharmacological interventions, 8 used only the Sleep Disturbance Scale in Children (SDSC), 4 used only the Pediatrics Sleep Questionnaire (PSQ), 2 used only the Child Sleep Habits Questionnaire (CSHQ), 1 used both the SDSC and the CSHQ, 1 applied both the CSHQ and the Pittsburgh Sleep Quality Index (PSQI), 1 used both the SDSC and the PSQI, and 1 used a questionnaire developed by the authors of the research. These studies are detailed in Table 2 .

Regarding nonpharmacological sleep interventions, studies 28 31 attest their indication as auxiliary management to sleep medications. The development of a sleep routine with regular sleeping and calming behaviors, environmental changes at home (such as light, temperature, bedding, and sound), and the interventions designed to improve the knowledge and ability of the parents to solve problems are examples of nonpharmacological interventions. 28 Currently, there is little research on this topic; however, interventions such as intense light therapy, activity, massage, and behavioral interventions present consistent results and no adverse effects. 17 Recently, a review of the literature present effectiveness in the use of phototherapy in circadian rhythm disorders (such as a non-24-hour sleep-wake rhythm disorder) in visually-impaired children. 32

Regarding caregivers, three studies used different scales or questionnaires to evaluate SDs in children and caregivers: in the first study, the SDSC questionnaire was applied; in the second, the authors developed and used their own questionnaire; and in the third study, the CSHQ was used. To evaluate the sleep of caregivers, two studies applied the PSQI, while the other was based on a questionnaire developed by the authors of the research; no study used the PSQ. Data related to the health of caregivers were evaluated in two studies. One study applied a questionnaire developed by the authors covering general health questions. Another study used the Major Depression Inventory evaluation, in which the objective was to establish the clinical diagnosis of depression and determine its severity. Three studies applied sociodemographic questionnaires. Two studies described the main occupation of caregivers as housewives. 18 33

In three studies, caregivers filled out sleep questionnaires, and a correlation between the results and SDs of children with CP was found. 11 18 33 One article pointed out that 70% of children with CP were sharing the bed with their mother (main caregiver). Bed-sharing was significantly correlated with SDs in caregivers, but it was not correlated with SDs in children with CP. 17 Two studies pointed out the correlation between SDs in caregivers and bed-sharing. 11 18 In addition, a correlation between SDs in children (total score in the SDSC questionnaire, sleep initiation and maintenance disorders, and excessive sleepiness disorders) and SDs in caregivers was also found. 18

One study 11 reported a significant increase in health problems, headaches, and psychological exhaustion among caregivers of children with sleep problems; a significant increase in wakefulness and interrupted sleep of caregivers (mothers and fathers) was also reported. Factors such as obstructive sleep apnea, position, muscle pain, gastroesophageal reflux, and the need for hygiene have been correlated with nighttime wakefulness of caregivers. 11 33 Regarding maternal health, maternal SD scores have been correlated with maternal depression. 33 The perception of the health of mothers was worse compared to that of fathers, in relation to poorer quality of sleep, pain due to overwork, and headache, according to one study. 11

## DISCUSSION

The present review provided a comprehensive synthesis of the literature on the nature of SDs, the factors related to these disorders, their treatment, and their impact on the lives of caregivers.

### Cerebral palsy and sleep disorders

#### *Factors associated with sleep disorders*

Our findings support the associations between SDs and motor limitations, gastrointestinal and respiratory problems, behavior problems, cognitive deficit, the presence of epilepsy, and sleep routine. 3 5 19 33 34 35

Regarding behavioral difficulties (such as hyperactivity) and SDs, it is not clear from the literature whether one acts as a predisposing factor to the other or whether both feedback mechanisms work together. Jan et al. 8 found a relationship between behavioral difficulties and SDs, and the author were able to observe manifestations such as vivid dreams, frequent awakening from anxiety or fear when falling asleep, trouble sleeping, difficulty in returning to sleep, and bruxism. Furthermore, studies have analyzed the association of behavioral difficulties with the presence of pain as another risk factor for SD. Oberlander et al. 31 suggested that the presence of pain and of cognitive and behavioral problems interferes with the quality of sleep of these children, generating drowsiness throughout the day, apathy, and impaired attention, conditions that can negatively impact school performance and participation of these children. 8 34 36 Another factor commonly found in children with CP is pain due to changes in postural tone and movement limitations. Thus, pain has been associated with a worse level of functionality and a higher prevalence of SDs, which results in worse quality of life for these children. 24 36 37

It is important to mention that epilepsy is described as a risk factor for SDs, but, at the same time, sleep-related seizures are part of SDs themselves. The relationship regarding epilepsy, epileptic seizures, and convulsive episodes of a different nature and SDs is of mutual influence: a pattern of sleep fragmentation and decreased rapid eye movement (REM) sleep – especially if chronically manifested –, which is frequent in epilepsy, can predispose to SDs, especially disorders of initiating and maintaining sleep (DIMS) and disorder of arousal (DA). 38 39 40 Although persistent seizures have been reported as a more important risk factor than the use of antiepileptic drugs, 6 19 their use has been described as a risk factor for the following SDs: DA; change in sleep total scale score; sleep breathing disorders (SBDs); sleep-wake transition disorder (SWTD); and disorders of excessive somnolence (DES). 18 26 41

In the study by Blake et al., 30 whose objective was to determine the effect of sleep positioning systems, which are devices commonly recommended by health professionals for the reduction of painful nocturnal movement of pelvic bones in children with CP unable to walk, the authors reported limited results when evaluating the effectiveness of these systems on sleep quality and pain. This indicates that there were no significant changes in these health aspects when using these devices or not. Therefore, to recommend the prescription of sleep positioning systems, more rigorous research is needed to determine effectiveness, cost-effectiveness, and the likelihood of adverse effects.

Other studies carried out with children with CP found a relationship between motor impairment and SDs. In 2017, Bautista et al. 19 reported that children with functional severity (Gross Motor Function Classification System level V) tend to present of severity SD because there is a reduced ability to roll in bed and change may experience more waking, more pain, and be less able to respond to airway obstruction. In addition, children with spastic quadriplegia and dyskinetic CP and visual impairment presented DIMS due to a delayed surge in nocturnal melatonin. Corroborating these findings, Adiga et al. 18 observed that restricted movements due to contractures, spasticity and motor impairment, pain to due to spasticity, dental caries, and the use of orthoses, impact on the quality of sleep of these children and initiated some type of SD.

Our findings suggest that hyperbaric oxygen (HBO2) therapy showed satisfactory results for sleep quality in children with CP. 10 After ten sessions of HBO2 therapy, there was a reduction in irritability and easy awakening, and prolonged sleep. 10 However, the mechanism that cause the effect of HBO2 on sleep remains unclear. In addition, it is possible that HBO2 reduces spasticity and decreases chronic pain through the production of analgesia, factors which can interfere with sleep. 10 However, HBO2 therapy cannot yet be considered a safe and effective method. Evidence on HBO2 therapy and sleep in children with CP is still scarce.

#### *Impact on caregivers*

The literature reports that SDs in children with CP interferes with the sleep of caregivers. According to Wright et al., 42 caregivers of children between 1 and 16 years of age with physical disabilities reported significantly more sleep disorders: 57% in comparison to 14% reported by parents of nondisabled children in the same age group. 42 In a study of 2,830 Norwegian mothers caring for children aged 6 to 20 weeks, an average score of 6.3 was observed in the PSQI, while that of mothers of children with CP was of 5.5. 43 This data suggests that the levels of SDs of mothers (caregivers) of children with CP are similar to those of mothers who care for small babies. 33 43

Children with CP may present DIMS and excessive sleepiness, situations which can affect the quality of sleep of caregivers. 18 Another factor that directly impacts the sleep of caregivers is epilepsy: the seizures can impair the quality and restoration of sleep by modifying the circadian sleep-wake rhythms and total sleep duration. These situations can trigger frequent nocturnal awakenings and interfere with the sleep pattern of caregivers. 18 23

Bed-sharing and night watch contribute to the worsening of the sleep of caregivers. Bed-sharing occurs due to children's constant need for attention from their parents, such as the very consequence of SDs and decreased mobility in bed. 18 43 Children needing night watch present little mobility, pain, and respiratory and digestive problems. These findings are similar to those of Petersen et al., 23 who reported that children with CP were awakened due to night pain, restlessness, and the need for repositioning and hygiene care. For this reason, caregivers had to wake up during the night to provide assistance to children. Hemmingsson et al. 33 reported that 39% of children needed assistance one or more times every night, a situation that impaired the daytime life of 74% of the caregivers.

Sleep disorders in children with CP interfere with the health perception of caregivers. Headaches, depression, and psychological problems are detected more frequently among them. Research related to the quality of life of caregivers of children with CP reported that this population has a higher prevalence of fatigue, pain, functional deficits, SDs, social restrictions, and emotional problems when compared with mothers of children with typical development (healthy children). 43 High levels of depression in caregivers are related to the stress resulting from dealing with a chronic condition such as CP. 44 45 Caring for children with CP can be exhausting and challenging due to physical limitations, care needs, and mobility. 45 46 In addition, caregivers are responsible for organizing and coordinating medical and rehabilitation services, situations which end up overloading them. 22 47 48

### Limitations

The present review was not without limitations. It is important to highlight that the vast majority of the original studies analyzed had a level VI of evidence. Regarding the nonpharmacological intervention research, we found no specific approach to psychological or occupational therapy methodologies. Studies on the impact of SDs on the lives of caregivers are still limited and do not have a consistent level of evidence. Therefore, the present work does not exhaust all the lines of research in the field of SDs in CP, but aims at elucidating the current knowledge on the studied themes, which is important for public policies and for new, impacting research.

In conclusion, from the analysis of the present study, it was possible to find the highest prevalence of SDs in the population with CP and its negative impact on the quality of lives of their family members and caregivers, in addition to suggesting nonpharmacological strategies as a form of intervention for SDs. However, further studies with a higher level of evidence are warranted, especially those evaluating the long-term impact of SDs and the effectiveness of nonpharmacological treatments.
